# The Multiscale Dynamics of Beat-to-Beat Blood Pressure Fluctuation Links to Functions in Older Adults

**DOI:** 10.3389/fcvm.2022.833125

**Published:** 2022-02-23

**Authors:** Xin Jiang, Yurun Cai, Xiaoyan Wu, Baofeng Huang, Yurong Chen, Lilian Zhong, Xia Gao, Yi Guo, Junhong Zhou

**Affiliations:** ^1^Department of Geriatrics, Shenzhen People's Hospital, Shenzhen, China; ^2^The Second Clinical Medical College, Jinan University, Shenzhen, China; ^3^The First Affiliated Hospital, Southern University of Science and Technology, Shenzhen, China; ^4^Department of Epidemiology, Johns Hopkins Bloomberg School of Public Health, Baltimore, MD, United States; ^5^Department of Neurology, Shenzhen People's Hospital, Shenzhen, China; ^6^Shenzhen Bay Laboratory, Shenzhen, China; ^7^Hinda and Arthur Marcus Institute for Aging Research, Hebrew SeniorLife, Roslindale, MA, United States; ^8^Division of Gerontology, Beth Israel Deaconess Medical Center, Boston, MA, United States; ^9^Harvard Medical School, Boston, MA, United States

**Keywords:** beat-to-beat blood pressure fluctuation, multiscale entropy, functional independence, vessel function, blood lipids

## Abstract

**Background:**

The blood pressure (BP) is regulated by multiple neurophysiologic elements over multiple temporal scales. The multiscale dynamics of continuous beat-to-beat BP series, which can be characterized by “BP complexity”, may, thus, capture the subtle changes of those elements, and be associated with the level of functional status in older adults. We aimed to characterize the relationships between BP complexity and several important functions in older adults and to understand the underlying factors contributing to BP complexity.

**Method:**

A total of 400 older adults completed a series of clinical and functional assessments, a finger BP assessment of at least 10 min, and blood sample and vessel function tests. Their hypertensive characteristics, cognitive function, mobility, functional independence, blood composition, arterial stiffness, and endothelial function were assessed. The complexity of systolic (SBP) and diastolic (DBP) BP series was measured using multiscale entropy.

**Results:**

We observed that lower SBP and DBP complexity was significantly associated with poorer functional independence (β > 0.17, *p* < 0.005), cognitive function (β > 0.45, *p* = 0.01), and diminished mobility (β < −0.57, *p* < 0.003). Greater arterial stiffness (β < −0.48, *p* = 0.02), decreased endothelial function (β > 0.42, *p* < 0.03), and excessed level of blood lipids (*p* < 0.03) were the main contributors to BP complexity.

**Conclusion:**

Blood pressure complexity is closely associated with the level of multiple functional statuses and cardiovascular health in older adults with and without hypertension, providing novel insights into the physiology underlying BP regulation. The findings suggest that this BP complexity metric would serve as a novel marker to help characterize and manage the functionalities in older adults.

## Introduction

Cardiovascular health is critical to maintaining functional independence in everyday activities in older adults. The conditions in the cardiovascular system have been linked to diminished functionalities. Hypertension, for example, a condition related to abnormal blood pressure (BP) regulation, is highly prevalent in older adults (1) and has been closely linked to slowed walking speed, cognitive impairment, and risk events (e.g., falls) in older adults (2–4). Therefore, characterizing the BP regulation and understanding the pathway through which BP regulation is associated with those functions will ultimately help optimize the management of cardiovascular health and important functional performance in older adults.

The BP is determined by multiple elements, such as the cardiac output and the systemic vascular resistance, and is regulated continuously by underlying neural and hormonal feedback procedures in neurovascular systems, including baroreceptors, resistance vessels, and sympathetic and parasympathetic nervous systems, over *multiple, not single*, scales of time (5, 6). Similar to the regulation of other neurophysiological procedures (e.g., heartbeat, the resting-state brain activities), the multiscale dynamics of continuous beat-to-beat BP fluctuation are “complex,” containing rich physiologically meaningful information pertaining to those underlying elements and their interactions (7, 8). The traditional widely used BP metrics, such as the mean level and/or variability of BP, are based upon one *single scale* of BP fluctuation, and may thus not appropriately characterize such physiologically complex multiscale dynamics/patterns pertaining to the regulation of BP (7, 8).

Recently, studies have emerged to quantitatively measure the multiscale dynamics of beat-to-beat BP fluctuation using “complexity,” a concept derived from the theories of the complex system. Several non-linear dynamic analytical techniques have been used to quantify the complexity in the output signals of human neurophysiological procedures including BP fluctuation. Studies have observed that, for example, the complexity of beat-to-beat BP fluctuations (i.e., BP complexity), as quantified using multiscale entropy (MSE) (9), is associated with vascular functions, while traditional single-scale metrics are not (7, 8, 10–12). In a series of our recent work, for example, lower complexity of the beat-to-beat BP fluctuation was associated with hypertension, greater severity of white matter lesions, and slower walking speed (7, 8), and was linked to increased risk of dementia in older adults (11). These observations suggest that the degree of BP complexity may be a marker for the subtle changes in the cardiovascular system, capturing the pathology in those age-related diseases and functional decline. However, the relationship between BP complexity and multiple important functions (e.g., capacity to complete daily activities independently, mobility), as well as the underlying characteristics within the vascular circulation, including blood composition (e.g., blood lipids) and vascular function (e.g., arterial stiffness), in older adults, has not been examined well.

In this study, we assessed multiple functions (e.g., functional independence) in a group of older adults, measured their BP complexity, and assessed the characteristics of blood samples and vessels. We hypothesized that: (1) lower BP complexity would be associated with poorer functions and (2) with the alterations in blood composition (e.g., increased blood lipids) and in vessel function (e.g., elevated arterial stiffness), suggesting the disruption in multiscale dynamics of BP fluctuation.

## Methods

### Participants

The participants were recruited *via* the data repository in the Department of Gerontology, Shenzhen People's Hospital, Shenzhen, China. Older adults who had a clinical visit for primary care/annual physical examination within the previous year and expressed interests in participating in future studies were contacted. The inclusion criteria were: (1) age greater than 60 years and (2) with the ability to walk for at least 1 min without physical assistance. The exclusion criteria were: (1) inability to understand the study protocol; (2) the diagnosis of terminal disease (e.g., cancer); (3) the diagnosis of overt neurological diseases (e.g., Parkinson's disease, stroke); (4) history of brain trauma or injury; (5) hospitalization within past 6 months; (6) uncontrolled hypertension; (7) chronic kidney disease and dyslipidemia; and (8) other cardiovascular diseases (e.g., heart failure, coronary artery disease) or targeted organ damage. All the experimental methods and protocols were approved by the Institutional Review Board of Shenzhen People's Hospital and carried out in accordance with the guidelines in the Declaration of Helsinki. All the participants provided a written consent in order to participate in this study.

### Study Protocol

After the screening, a total of 400 eligible older adults were eligible for the following tests. Each eligible participant completed two study visits separated by one day. On the first visit, they completed a series of questionnaires to assess the clinical characteristics, the functional dependence of daily activities, the Mini-Mental State Examination (MMSE) for general cognitive function, and the timed-up-and-go (TUG) test for mobility. On the second study visit, they completed the assessment of continuous BP, the blood sample test, and the assessment of arterial stiffness and vascular endothelial function. One study personnel monitored all the procedures for each visit to ensure the safety of participants.

#### Characteristics Related to Hypertension

Hypertension is the main condition in this population and affects BP regulation. We thus here carefully assessed the characteristics related to hypertension following the clinical diagnosis of each participant. Specifically, hypertension was characterized as the systolic BP (SBP) ≥ 140 mm Hg and/or diastolic BP (DBP) ≥ 90 mm Hg by measuring the brachial artery of the right arm using a digital sphygmomanometer (Omron, Kyoto, Japan). The duration of hypertension (i.e., history of hypertension) was recorded. In hypertensive participants, hypertension had been controlled using the antihypertension medication. The antihypertension medication consisted of calcium channel blockers (CCBs), angiotensin-converting enzyme inhibitors (ACEI), angiotensin-II receptor blockers (ARB), beta-blockers (BBs), and diuretics. The number of participants using each type of medication, and the number of antihypertensive medications each participant used at the same time were recorded.

#### Activities of Daily Living (ADL)

Barthel index for activities of daily living (ADL) (13) was used to assess the ADL in each participant. The maximum total score was 100 and the lower score reflected poorer functional independence in daily living activities.

#### Mini-Mental State Examination

The Chinese version of MMSE (14, 15) was used to assess the general cognitive function of each participant. The total score of MMSE was used in the analysis and a lower score reflected poorer cognitive function.

#### Mobility

The mobility of participants was assessed using a timed-up-and-go test (TUG). The TUG measures the time taken to stand from a chair, walk forward three meters, turn around, walk back, and return to a seated position (16). The time to completed TUG has been linked to mobility and is predictive of future falls in older adults (17). Each participant completed two TUG trials (separated by 30 seconds of rest), and the averaged TUG time across the two trials was used in the following analysis.

#### Blood Sample Test

Participants completed the blood sample test in the early morning of the second visit. They were asked to not eat before the visit. One experienced and trained nurse dropped the blood of 3 milliliters from the left arm of each participant when he/she was sitting in the chair. Multiple blood composition metrics, including those related to blood lipids [i.e., total cholesterol (CHOL), low- (LDL) and high- (HDL) density lipoprotein, and triglyceride (TG)], blood protein [i.e., hemoglobin (HGB), total protein (TP), and albumin (ALB)], blood sugar [i.e., glucose (GLU)], and the kidney function [i.e., creatinine (Cr), blood urea nitrogen (BUN), and uric acid (UA)], were analyzed by two analyzers (i.e., Beckman Coulter AU5800, CA and Sysmex XN-3000, Japan). We then constructed a composite score of blood lipids using CHOL, HDL, and LDL. We also calculated the BUN/Cr ratio, an important marker for kidney function (18). Both the composite score of blood lipids and BUN/Cr ratio were used in the following analyses in addition to each of the blood metrics.

#### Beat-to-Beat BP Recordings

**Figure 1:**
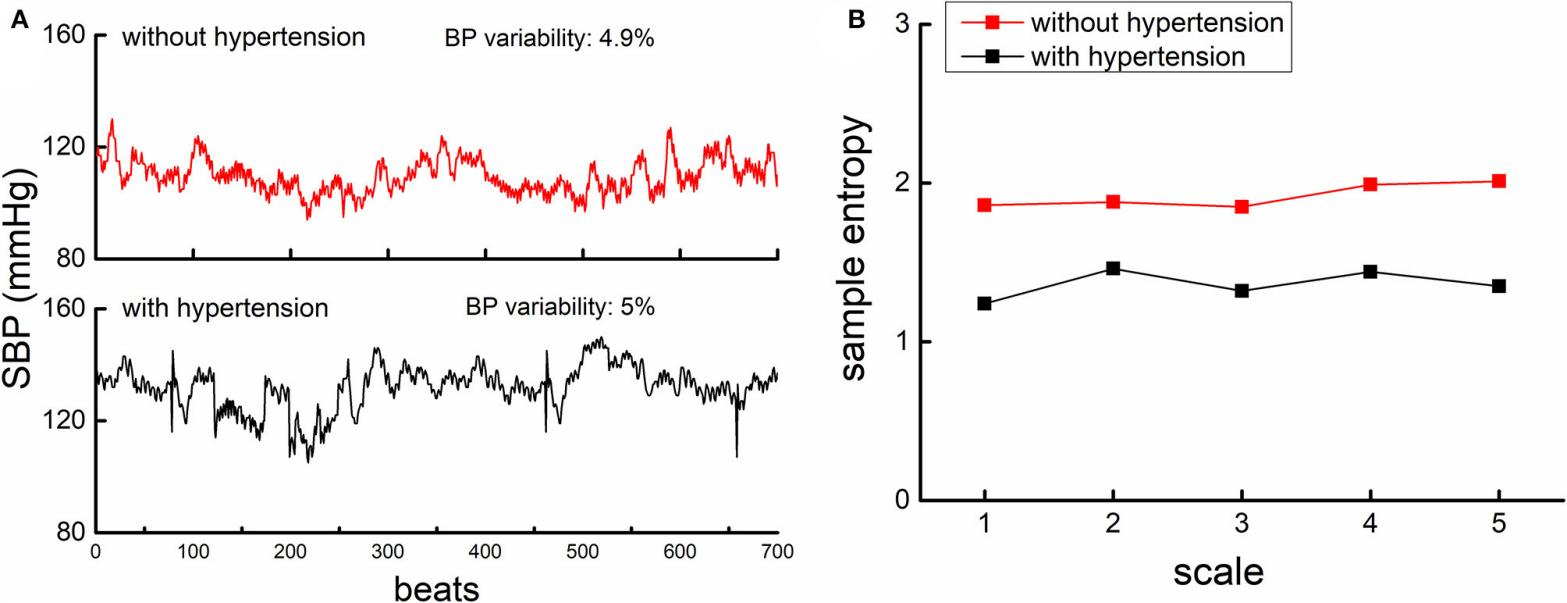
The beat-to-beat SBP series **(A)** and MSE curves **(B)** from one participant with hypertension (lower panel of A, black) and another participant without hypertension (upper panel of A, red). **(A)**. An example of the beat-to-beat SBP series from one participant without hypertension (upper left panel) and from one participant with hypertension (lower left panel). Both participants had similar BP mean level and BP variability as displayed in the figure. **(B)** shows the MSE curve calculated from the SBP series of these two participants. The entropies of the participant with hypertension were lower than that of participants without, across all the five scales. This suggested that the complexity of SBP fluctuation was lower in hypertensive as compared to non-hypertensive, while the BP variability was similar.

After at least 30 min of resting, each participant then completed a BP assessment when sitting in a quiet assessment room with one study staff. During the BP assessment, the participant was instructed to not talk and to keep motionless (e.g., not moving the arms). The objects which may interfere with the testing, such as the mobile phone, were saved outside the room. The continuous SBP and DBP series were recorded using the Finometer PRO system (Finapres Medical Systems BV, the Netherlands) at the middle finger of the left hand in a supine position for 10 to 15 min. The sampling frequency was 100 Hz (19). All the BP recordings consisted of at least 700 continuous beats, which enables reliable estimation of MSE (9) (see more details in BP complexity section later). The BeatScope software package (Finapres Medical Systems BV, the Netherlands) was then used to calculate the BP values of each beat. The outliers in the beat-to-beat BP series of which the value was greater or lower than the mean ± two SD of the series were interpolated by the mean (7, 8, 11). The preprocessed BP series of 700 sampling points (Figure 1A) were then used in the calculation of BP complexity.

#### Blood Pressure Complexity

The complexity of SBP and DBP series was quantified using MSE, a well-developed and widely used technique that quantifies the entropy or recurrence in physiologic series over different temporal or spatial scales. Specifically, the BP series was “coarse-grained” for scales from 1 to 5, i.e., the original series was divided into non-overlapping windows of length equaling to 1 to 5 sampling points. Thus, in the coarse-graining process, the series at Scale 1 was the raw series consisting of 700 data points, that at Scale 5 was constructed by averaging every five non-overlapped points, consisting of 140 points (i.e., 700 points/5). The sample entropies of each “coarse-grained” series were then calculated by using the negative natural logarithm of the conditional probability that a time series, having repeated itself within a tolerance r for m points (pattern length), will also repeat itself for m + 1 points without self-matches (9, 20, 21). We followed the same procedure of MSE calculation in our previous studies (7, 8) by choosing the parameter of tolerance r = 0.15 and the number of matching points m = 2. The number of points in the coarse-grained BP series at Scale 5 (*n* = 140) was greater than that is recommended for reliable estimation of sample entropy with the parameters we used (i.e., the number of data points in the coarse-grained time-series at the largest scale should be at least 10^m^ to 17^m^; here we chose m = 2, so the recommended number was 100–289) (9). BP complexity was then defined as the averaged entropy across five scales. Lower averaged MSE reflected lower complexity (Figure 1B).

In addition, the mean and variability (i.e., the coefficient of variation, the ratio of SD to the mean of the beat-to-beat BP series) of beat-to-beat SBP and DBP were calculated and used in the following analyses.

#### Assessment of Arterial Stiffness and Vascular Endothelial Function

Following the standard testing procedures as suggested in the previous studies (22–24), the arterial stiffness was assessed by measuring the left- and right-side brachial-ankle pulse wave velocity (baPWV) (Omron, Kyoto, Japan) and the vascular endothelial function was assessed by measuring the flow-mediated dilation (FMD) (Unex, Nagoya, Japan) when participants were in the resting state following the testing procedure. In addition to each metric, the composite score of vessel function was constructed and used in the analysis.

### Statistical Analysis

Statistical analyses were performed with JMP Pro. 15 software (SAS Institute, Cary, North Carolina, USA) and Mplus 8.4 (Muthén & Muthén, Los Angeles, California, USA). Data distribution and missing values were checked for all the variables. The significance level was set at *p* < 0.05.

We first examined the effects of hypertension on demographics, functionalities, vascular function, BP complexity, mean, and variability of BP by using ANOVA models. The factor of each model was hypertensive status (i.e., normotensive and hypertensive), and the dependent variable was each of those outcomes.

Then to examine the relationships between BP complexity (i.e., SBP and DBP complexity) and the functionalities, linear regression models were used. The relationship of BP complexity with functional independence in daily activities (i.e., ADL), mobility (i.e., TUG time), and cognitive function (i.e., MMSE score) was examined in the separate models. Similarly, linear regression analyses were also used to examine the relationship between traditional BP measures (i.e., the mean and variability of BP) and those functional characteristics. Age, sex, body mass index (BMI), the status of regular use of alcohol (i.e., alcoholic and non-alcoholic) and smoking (i.e., smoker and non-smoker) and hypertensive status (i.e., hypertensive and normotensive), the use of antihypertension medication, and the duration of hypertension were included as covariates in these regression models. Secondarily, within the hypertensive group, we also explored the relationship between BP complexity and the duration of hypertension using the regression model, in which age, sex, BMI, smoking, and alcohol status were included as covariates.

To examine the relationship between characteristics of blood lipids and vessel and BP complexity, we first used confirmatory factor analysis (CFA) with maximum likelihood estimation to derive the separate composite scores for blood lipids and vessels. Each composite score had a mean of 0 and a variance of 1. The relationships between each of the composite scores and BP complexity were examined using separate regression models. We then further used separate linear regression models to explore the relationship of BP complexity with each metric of blood composition related to proteins, lipids, and kidney function, and the metrics of vessel function. All these models were adjusted for age, sex, BMI, the status of regular use of alcohol (i.e., alcoholic and non-alcoholic) and smoking (i.e., smoker and non-smoker), and hypertensive status (i.e., hypertensive and normotensive), the use of antihypertension medication, and the duration of hypertension. To enhance the interpretability of the regression coefficients, BP complexity was standardized for models.

## Results

All the participants completed the tests of this study. A total of 247 participants were identified as hypertensive and the other 153 were normotensive. Table 1 shows the demographics and clinical and functional characteristics of the entire population and within hypertensive and normotensive groups. Compared to the normotensive group, the hypertensive group had older age, poorer cognitive function (i.e., lower MMSE score), higher left and right baPWV, lower FMD, the greater level of Cr, BUN, UA, CHOL, LDL, and GLU in the blood, and higher mean BP and BP variability (*p* < 0.03). Meanwhile, it was also observed that compared with the normotensive, the hypertensive group had significantly lower BP complexity (*p* < 0.004), which was consistent with our previous studies (7, 8). No significant association between BP complexity and the mean and variability of beat-to-beat BP fluctuation was observed (β = −0.1 to 0.1, *p* = 0.28–0.55).

**Table 1:** Demographics and clinical information, characteristics of blood pressure, blood composition, and vessel function in participants.

**Mean ± S.D**.		**Total** **(*n* = 400)**	**Hypertensive group** **(*n* = 247)**	**Normotensive group** **(*n* = 153)**	***P* value**
Age (years)		71.2 ± 8.2	72.2 ± 8.2	69.8 ± 8	0.01
Sex (*n* = female)		179	105	74	0.84
BMI		24.2 ± 3.6	24.7 ± 3.5	23.3 ± 3.5	0.22
MMSE total score		25.5 ± 4.8	24.8 ± 5.2	26.5 ± 4.1	0.006
TUG (s)		13.9 ± 6.5	14.8 ± 7.2	12.4 ± 4.8	0.003
Smoking (*n* = smokers)		49	27	22	0.87
Alcohol (*n* = alcoholic)		9	7	2	0.19
Total score of ADL		96.4 ± 10	95.8 ± 11.4	97.5 ± 7.1	0.22
PWV (m/s)	Left	13.8 ± 4.4	14.5 ± 4.7	12.7 ± 3.4	<0.001
	Right	13.5 ± 3.9	14.2 ± 4.2	12.3 ± 3.2	<0.001
FMD (%)		3.5 ± 2.2	3.2 ± 1.9	3.8 ± 2.5	0.03
Mean BP level	SBP	133.6 ± 15.7	140.3 ± 15.6	122 ± 13.2	<0.001
	DBP	77.7 ± 9.9	78.2 ± 10.4	75.6 ± 8.8	0.001
BP variability (%)	SBP	6 ± 1.7	6.8 ± 1.5	5.9 ± 1.6	0.01
	DBP	3.4 ± 1.4	3.8 ± 1.5	3.2 ± 1.2	0.02
BP complexity	SBP	1.3 ± 0.3	1.4 ± 0.2	1.5 ± 0.3	0.004
	DBP	1.4 ± 0.3	1.3 ± 0.3	1.4 ± 0.3	0.001
Duration of hypertension			11.4 ± 8.3	N.A	N.A
Anti-hypertensive medication (n)	CCB		109	N.A	N.A
	ACEI		13	N.A	N.A
	ARB		67	N.A	N.A
	BB		56	N.A	N.A
	Diuretics		20	N.A	N.A
Number of medications	1		81	N.A	N.A
	2		52	N.A	N.A
	3		23	N.A	N.A
	4		5	N.A	N.A
	5		1	N.A	N.A
HGB (g/L)		130.6 ± 17.1	131.7 ± 18.5	130.4 ± 14.9	0.59
TP (g/L)		66.8 ± 5.9	66.7 ± 6.6	66.9 ± 4.5	0.93
ALB (g/L)		411.6 ± 3.9	41.5 ± 3.9	41.5 ± 3.8	0.72
Cr (umol/L)		81.1 ± 27.9	85.2 ± 31.3	74.4 ± 19.6	<0.001
BUN (mmol/L)		5.7 ± 2.3	6 ± 2.7	5.2 ± 1.5	0.003
BUN/Cr ratio		71.3 ± 18.7	70.7 ± 19.1	72.3 ± 18.2	0.43
UA (umol/L)		348.3 ± 91.3	358 ± 93	331.1 ± 85.8	0.008
GLU (mmol/L)		6.3 ± 2.5	6.6 ± 2.8	5.7 ± 1.6	0.001
CHOL (mmol/L)		4.5 ± 1.2	4.4 ± 1.1	4.7 ± 1.3	0.01
LDL (mmol/L)		2.6 ± 1.0	2.5 ± 0.9	2.7 ± 1.0	0.02
HDL (mmol/L)		1.2 ± 0.3	1.2 ± 0.3	1.2 ± 0.3	0.22
TG (mmol/L)		1.5 ± 1.0	1.5 ± 1.1	1.4 ± 0.5	0.37

*BMI, body mass index; FMD, Flow mediated dilation; PWV, pulse wave velocity; HGB, hemoglobin; TP, total protein; ALB, albumin; Cr, creatinine; BUN, blood urea nitrogen; UA, uric acid; GLU, glucose; CHOL, cholesterol; LDL, low-density lipoprotein; HDL, high-density lipoprotein; TG, triglyceride*.

### Relationship Between BP Complexity and Functions in Older Adults

**Figure 2:**
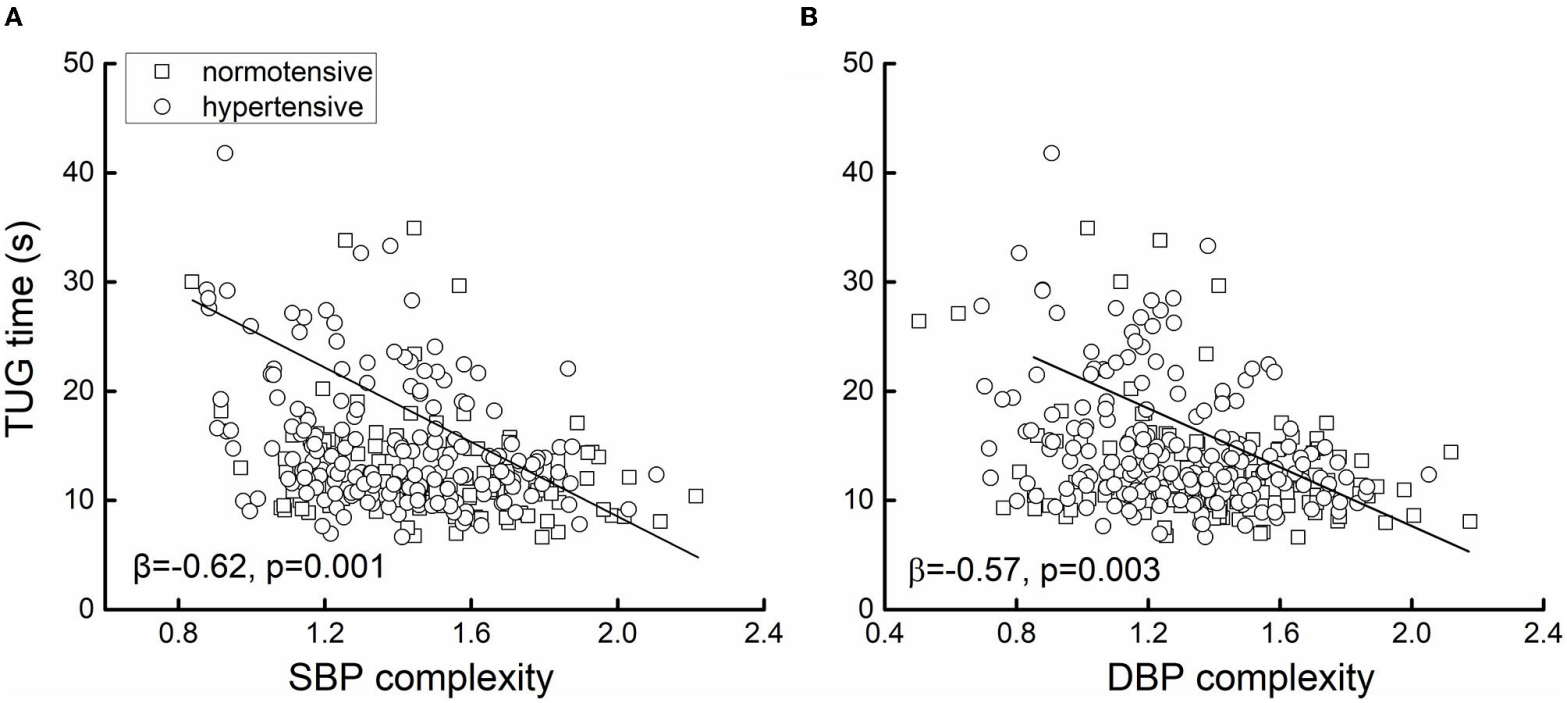
The association between systolic blood pressure (SBP) **(A)** and diastolic blood pressure (DBP) **(B)** complexity and mobility. The mobility was measured by the time to complete the timed-up-and-go test (i.e., TUG time). Greater TUG time reflected poorer mobility. Linear regression analyses adjusted for age, sex, body mass index (BMI), alcoholic and smoking status, hypertension, the use of antihypertension medication, and the duration of hypertension showed that in the 400 older adults, lower SBP (β = −0.62, *p* < 0.0001) and/or DBP (β = −0.57, *p* = 0.003) complexity was associated with greater TUG time (i.e., worse mobility). Different markers on the figure presented the hypertensive status [i.e., hypertensive (*n* = 247) or normotensive (*n* = 153)].

Figure 1 shows the example of the beat-to-beat BP series (A) and the MSE curve (B) from one participant in the hypertensive group and the other in the normotensive group. The BP variability was similar between them, but the hypertensive individual had much lower BP complexity than the normotensive individual. Linear regression analyses revealed that SBP and DBP complexity was significantly associated with TUG time (SBP: β = −0.62, *p* = 0.001; DBP: β = −0.57, *p* = 0.003; Figure 2), ADL score (SBP: β = 0.18, *p* = 0.002; DBP: β = 0.17, *p* = 0.005), and MMSE score (SBP: β = 0.45, *p* = 0.01; DBP: β = 0.48, *p* = 0.01) (Table 2). Specifically, participants with lower SBP and/or DBP complexity had longer TUG time (i.e., poorer mobility), lower ADL score (i.e., lower independence in daily living), and lower MMSE score (i.e., poorer cognitive function) (Table 2). No such significant association between the mean and variability of BP and these functions was observed (β = −0.03–0.03, *p* = 0.21–0.91) (Table 2). All these relationships were independent of age, sex, BMI, hypertensive status (i.e., hypertensive and normotensive), the use of antihypertension medication, duration of hypertension, and regular use of alcohol and smoking (i.e., smoker and non-smoker). Secondarily, we observed that within the hypertensive group, those with a longer duration of hypertension had significantly lower SBP (β = −0.3, *p* = 0.01) and/or DBP (β = −0.25, *p* = 0.01) complexity, and such relationships were independent of age, sex, BMI, smoking, and alcohol status.

**Table 2:** The association between blood pressure (BP) metrics and functions in the older adults.

**β value**		**TUG time**	**ADL score**	**MMSE score**
SBP	Mean	−0.01	0.02	0.02
	Variability	−0.02	0.03	−0.03
	Complexity	−0.62^**^	0.18^**^	0.45^*^
DBP	Mean	0.01	−0.03	−0.02
	Variability	0.02	0.03	0.01
	Complexity	−0.57^**^	0.17^*^	0.48^*^

* *Indicated p < 0.05;*

** *indicated p < 0.005. All the regression models were adjusted for age, sex, BMI, hypertensive status (i.e., hypertensive and normotensive), the use of antihypertension medication, duration of hypertension, and status of regular use of alcohol and smoking (i.e., smoker and non-smoker)*.

### Relationship Between BP Complexity, Blood Composition, and Vessel Characteristics

In unadjusted models, the composite score of vessel characteristics had a significant association with SBP (β = −0.30, *p* < 0.001) and DBP (β = −0.29, *p* < 0.001) complexity. After adjusting for covariates, the association was attenuated but remained significant for both SBP (β = −0.17, *p* = 0.04) and DBP complexity (β = −0.18, *p* = 0.03) (Table 3). Meanwhile, the composite score of blood lipids were not statistically associated with either SBP or DBP complexity (β < 0.09, *p* > 0.05).

**Table 3:** The relationship of BP complexity with vessel function and blood composition.

**β value**		**SBP complexity**	**DBP complexity**
Composite scores	Vessel characteristics	−0.17^*^	−0.18^*^
	Blood lipids	−0.1	0.09
	Blood protein	0.05	0.08
Left PWV		−0.51^*^	0.42^*^
Right PWV		−0.5^*^	−0.48^*^
FMD		0.55^*^	0.42^*^
CHOL		−0.47^*^	−0.42^*^
HDL		−0.48^*^	−0.41^*^
LDL		0.49^*^	0.42^*^
TG		−0.24^*^	−0.24^**^
BUN/Cr		−0.22^*^	−0.26^**^
HGB		0.09	0.08
TP		0.01	0.03
ALB		0.04	0.06
GLU		0.07	0.08

* *Indicated p < 0.05;*

** *indicated p < 0.005. All the regression models were adjusted for age, sex, BMI, hypertensive status (i.e., hypertensive and normotensive), the use of anti-hypertension medication, duration of hypertension, and status of regular use of alcohol and smoking (i.e., smoker and non-smoker)*.

We then examined the relationship between each metric of blood composition vessel function and BP complexity. Linear regression analyses adjusted for age sex, BMI, hypertensive characteristics, and status of taking alcohol regularly and smoking showed that the SBP and DBP complexity was significantly associated with the left (SBP: β = −0.51, *p* = 0.02; DBP: β = −0.42, *p* = 0.01) and right (SBP: β = −0.5, *p* = 0.02; DBP: β = −0.48, *p* = 0.02) baPWV. Participants with greater baPWV (i.e., worse arterial stiffness) had lower complexity of SBP and/or DBP. A significant association between FMD and BP complexity was also observed, that is, participants with lower FMD had lower SBP (β = 0.55, *p* = 0.02) and/or DBP (β = 0.42, *p* = 0.03) complexity (Table 3). The linear regression models also showed that several metrics related to blood lipids were associated with BP complexity. Specifically, participants with lower HDL (SBP: β = 0.49, *p* = 0.02; DBP: β = 0.42, *p* = 0.01), higher LDL (SBP: β = −0.48, *p* = 0.02; DBP: β = −0.41, *p* = 0.02), CHOL (SBP: β = −0.47, *p* = 0.02; DBP: β = −0.42, *p* = 0.03), and/or TG (SBP: β = −0.24, *p* = 0.01; DBP: β = −0.24, *p* = 0.003) had lower BP complexity (Table 3). Those with higher BUN/Cr ratio (i.e., related to worse kidney function) also had significantly lower SBP (β = −0.22, *p* = 0.02) and/or DBP (β = −0.26, *p* = 0.002) complexity. No significant association was observed between BP complexity and other blood composition metrics (e.g., blood proteins), and between the mean BP level and the blood composition metrics (β = 0.01–0.09, *p* = 0.21–0.89, Table 3).

## Discussion

We here demonstrated for the first time that the multiscale dynamics of continuous SBP and DBP fluctuations, as quantified by “BP complexity,” is associated with multiple important functional performances including the mobility, cognitive function, and functional independence in daily activities in older adults with and without hypertension. Meanwhile, for the first time, we observed the association between the blood sample composition, vessel and vascular endothelial function, and BP complexity, providing novel knowledge into the underlying cardiovascular elements that contribute to the regulation of BP fluctuation.

Inconsistent with previous studies (7, 8, 10–12), we here observed significant associations between BP complexity and several important functions in the older adults. The theory of complexity in aging tells that aging and age-related conditions often alter the quantity and/or quality of the biophysiological elements in a given physiologic system and their interactions over multiple temporospatial scales. Such age-related alterations can often be captured by the disrupted multiscale dynamics (i.e., loss of complexity) in the fluctuations of the spontaneous output signals of this system (25). Mounting evidence has suggested that compared to traditional single-scale measures (e.g., mean or variability), the complexity measures of the physiologic fluctuations can provide more insights into the multiscale nature of the regulation in the physiological procedures (7, 8, 10, 21, 26–30). Numerous studies have linked the complexity metric to important functional performance and to the age- and disease-related subtle changes in physiologic systems, including the heartbeat series, standing postural sway fluctuation, brain activities as measured by electroencephalogram and functional MRI (7, 8, 10, 21, 26–30). For example, Rangasamy et al. previously observed that lower preoperative BP complexity was associated with greater frailty in older adults after surgery (10). Here, we observed in a group of older adults with a much larger sample size that lower complexity of the beat-to-beat continuous BP fluctuation is associated with the poorer functional independence in daily activities (i.e., lower ADL score), poorer cognitive function (i.e., lower MMSE score), and diminished mobility (i.e., longer time to complete TUG). Taken together, this study provides confirmatory but novel evidence that BP complexity captures the changes in the regulation of the cardiovascular system pertaining to the level of important functional status in older adults.

This is the first study to explore the potential factors within the blood composition and vessel function that may contribute to the multiscale dynamics of BP fluctuation. It is observed that the metrics of blood lipids are significantly associated with the level of BP complexity across older adults with and without hypertension, though the composite score of blood lipids we constructed is not; and both the composite score and specific metrics of vessel function are significantly associated with the level of BP complexity. Older adults with a lower level of HDL, higher level of LDL, CHOL, and/or TG, and those with greater arterial stiffness (i.e., higher baPWV) and/or diminished endothelial function (i.e., lower FMD) had lower BP complexity, suggesting the disrupted patterns of the BP fluctuations. Blood lipids are important to our vascular circulation system. For example, cholesterol is needed to build artery walls and to make hormones and vitamin D (31), and the lipoproteins carry cholesterol and triglycerides circulating in the blood (32). However, the excess of the cholesterol and triglycerides, and also the LDL (i.e., “bad” lipoproteins), may develop fatty deposits in blood vessels, block the blood flow; while the HDL (i.e., “good” lipoproteins) helps absorb the cholesterol and carries it back to the liver to flush the cholesterol from the body, helping the blood flow and supply in the vascular system (33, 34). Therefore, a lower level of HDL, but a higher level of LDL, CHOL, and TG, may alter the normal circulation in the vascular system, altering the cardiovascular regulation of BP. Meanwhile, studies have shown that both the increased stiffness of the artery and the endothelial dysfunction induce multiple functional impairments in the cardiovascular system, such as the diminished compliance of the vessel (35–38). Such functional decline may severely alter the contraction and dilatation of the vessel. Taken together, both alterations in blood lipids and vessel characteristics, which disrupt the BP regulation, are related to diminished BP complexity. Future studies are required to characterize other dysfunction or impairment in the vascular system, such as the target organ damage (TODs) (39, 40), and examine how these dysfunctions contribute to BP complexity. This will help future characterize the biophysiological procedures underlying the regulation of BP.

On the other hand, the alterations in the characteristics of blood lipids and vessels are highly prevalent in the older adults with hypertension and have been closely linked to the risk of cardiovascular events (e.g., atherosclerosis, heart failure) (37, 41, 42). Ohkuma et al., for example, observed that the baseline baPWV in older adults without cardiovascular disease (CVD) is associated with the risk of development of CVD in the follow-up 6 years and a half. The hazard ratio for CVD was 3.5 times more in those in the highest quintile of baPWV as compared to those in the lowest quintile of baPWV (42). The observed association between BP complexity and the level of blood lipids, arterial stiffness, and endothelial dysfunction across older adults with and without hypertension may thus, in turn, indicate that BP complexity would also potentially be linked to these cardiovascular risks, which needs to be explored in future studies with a longitudinal design.

Another significant pathological condition in older adults is the kidney disease, which can be assessed by an abnormal level of BUN, Cr, or UA in blood. More recently, studies have shown that the ratio of BUN to Cr (i.e., BUN/Cr ratio) is sensitive to the renal dysfunction (43, 44). In this study consisting of older adults without any chronic renal disease or conditions, compared to the normotensive group, hypertensive participants have a significantly greater level of BUN, Cr, and UA, but no significant difference is observed in the BUN/Cr ratio, which is within the normal range (i.e., 20–100). However, even in these relatively “renal-healthy” individuals, lower BP complexity is significantly associated with a greater BUN/Cr ratio, indicating the BP complexity may also capture the subtle changes within the vascular circulation underlying the pathology of kidney diseases or conditions.

One important limitation is that this study only examined the cross-sectional relationship between BP complexity and functional status. Therefore, future longitudinal studies are highly demanded to examine the casual relationship between BP complexity and the changes in those functional statuses and the pathology of the conditions related to abnormal regulation of BP (e.g., kidney disease). The use of antihypertension medication in the hypertensive group may potentially contribute to BP complexity and other functions. Though we included the medication information in our analyses, we here did not explicitly examine the effects of medication on BP complexity, and on the relationship between BP complexity and functions because of the uneven number of medications (e.g., only 23 participants simultaneously used three types of antihypertensive medications, while 81 participants used only one type of medication). Therefore, it is worthwhile to explore how the use of antihypertensive medication influences the regulation of BP and its relationship with important functions in older adults in future studies. The BP was measured only in a short term at resting state in this study. While this suggests that this BP complexity metric can be obtained conveniently, previous studies, on the contrary, showed that the ambulatory BP across hours or days was also associated with important functional status (e.g., cognitive function) in older adults (45, 46). It is thus worthwhile to characterize the BP complexity of the ambulatory BP recordings over larger scales. Nevertheless, the findings of this study demonstrate that BP complexity as quantified using short-term continuous BP recording may capture the subtle changes within cardiovascular systems that are pertaining to the level of several important functional statuses in the older adult population, holding great promise to help the assessment and management of cardiovascular health in near future.

## Data Availability Statement

The raw data supporting the conclusions of this article will be made available by the authors, without undue reservation.

## Ethics Statement

The studies involving human participants were reviewed and approved by the Institutional Review Board of Shenzhen People's Hospital. The patients/participants provided their written informed consent to participate in this study.

## Author Contributions

XJ, YCa, YG, and JZ contributed to study conception and design. XW, BH, YCh, LZ, and XG contributed to data collection. XJ, YCa, XW, BH, YCh, LZ, XG, YG, and JZ contributed to data acquisition, analysis, and interpretation of results. XJ, YCh, YG, and JZ drafted the manuscript. All authors contributed to the article and approved the submitted version.

## Funding

This study was supported by Sustainable Development Science and Technology Project of Shenzhen Science and Technology Innovation Commission (KCXFZ20201221173411032 and KCXFZ20201221173400001), the Basic Research Project of Shenzhen Natural Science Foundation, the Shenzhen Science and Technology planning project (JCYJ20190807145209306), the Natural Science Foundation of Guangdong Province (2021A1515010983), and the Shenzhen Key Medical Discipline Construction Fund (Nos. SZXK012 and SZXK005).

## Conflict of Interest

The authors declare that the research was conducted in the absence of any commercial or financial relationships that could be construed as a potential conflict of interest.

## Publisher's Note

All claims expressed in this article are solely those of the authors and do not necessarily represent those of their affiliated organizations, or those of the publisher, the editors and the reviewers. Any product that may be evaluated in this article, or claim that may be made by its manufacturer, is not guaranteed or endorsed by the publisher.
